# Sense classification of shallow discourse relations with focused RNNs

**DOI:** 10.1371/journal.pone.0206057

**Published:** 2018-10-30

**Authors:** Gregor Weiss, Marko Bajec

**Affiliations:** University of Ljubljana Faculty of Computer and Information Science, Ljubljana, Slovenia; University of Bari, ITALY

## Abstract

Understanding the sense of discourse relations between segments of text is essential to truly comprehend any natural language text. Several automated approaches have been suggested, but all rely on external resources, linguistic feature engineering, and their processing pipelines are built from substantially different models. In this paper, we introduce a novel system for sense classification of shallow discourse relations (FR system) based on focused recurrent neural networks (RNNs). In contrast to existing systems, FR system consists of a single end-to-end trainable model for handling all types and senses of discourse relations, requires no feature engineering or external resources, is language-independent, and can be applied at the word and even character levels. At its core, we present our novel generalization of the focused RNNs layer, the first multi-dimensional RNN-attention mechanism for constructing text/argument embeddings. The filtering/gating RNN enables downstream RNNs to focus on different aspects of the input sequence and project it into several embedding subspaces. These argument embeddings are then used to perform sense classification. FR system has been evaluated using the official datasets and methodology of CoNLL 2016 Shared Task. It does not fall a lot behind state-of-the-art performance on English, the most researched and supported language, but it outperforms existing best systems by 2.5% overall results on the Chinese blind dataset.

## Introduction

To truly comprehend any natural language text, we need to interpret more than just the meaning of its parts. We need to infer additional semantic relations, known as discourse relations or coherence relations, that describe how different segments of text and abstract objects are related to each other. This is a open problem in many structure-enabled language applications [1], such as statistical machine translation [2], text summarization [3], sentiment analysis [4], question generation [5], coherence modelling [6], and discourse parsing [7].

We focus on the task of sense classification of shallow discourse relations as described in the CoNLL 2016 Shared Task [8], which is the most challenging part of such systems. It is formulated as follows: Given a piece of text with marked pair of arguments (arg1, arg2), a connective ($\underline{\underline{\text{conn}}}$), and punctuation (punc), our task is to predict the sense label, that relates both arguments (10 labels for Chinese and 21 for English).

1. *[Jane fell over]*_*arg1*_, $\underline{\underline{when}}$ *[Tarzan offered to help her]*_*arg2*_.
2. *[I’ll be there at nine]*_*arg1*_, $\underline{\underline{unless}}$ *[the train is late]*_*arg2*_.
3. *[I want to go home for the holidays]*_*arg1*_. $\underline{\underline{Nonetheless}}$, *[I will book a flight to Hawaii]*_*arg2*_.

Previous examples show a few Explicit discourse relations, where the sense is signalled by an overt discourse connective ($\underline{\underline{\text{when}}}$ signals a causal relation, $\underline{\underline{\text{unless}}}$ an alternative, and $\underline{\underline{\text{nonetheless}}}$ a comparison). From a computational perspective, it is relatively straightforward to predict these senses by concentrating on discourse connectives and carefully designing production rules to disambiguate their function [9]. However, more than half of the discourse relations in a text are not signalled by a discourse connective. Consider how previous examples change, if we transform them into Implicit discourse relations by dropping the underlined discourse connective (without $\underline{\underline{\text{when}}}$ the temporal ordering of events is reversed, without $\underline{\underline{\text{unless}}}$ or $\underline{\underline{\text{nonetheless}}}$ we infer a causal relation). In such situations the sense needs to be inferred through the semantic context, coherence of arguments, or other meaning [10]. From a computational perspective, such situations are much more challenging and represent a bottleneck of entire systems.

Most existing systems to tackle sense classification are complex and designed for the English language, they rely on linguistic feature engineering, external lexicons, syntactic parsers, and other resources. It is not obvious how to extend them to languages with a different grammar and structure, less linguistic resources, and different label sets, such as Chinese. To improve on this, conferences CoNLL 2015 and 2016 organized a Shared Task [8, 11] focusing on shallow discourse parsing on English and Chinese languages. Sense classification is still its most challenging part. Around half of the methods used conventional machine learning techniques such as MaxEnt, SVM and CRF models that rely on thousands to millions of hand-engineered features constructed from word categories and positions [9], production and dependency rules [12], neighbouring words, syntactic parse trees and part-of-speech (POS) tags [13], and cross-argument similarity features based on word pairs [14]. These generally make weak predictors of the relation sense, and increase the complexity of the solutions, but nevertheless work pretty good for Explicit relations. The other half used different neural network models and relied on pre-trained word embeddings combined with previously mentioned hand-engineered features. On word embeddings of each argument they separately apply either a variant of summation pooling [15], convolutional neural network [14, 16], or recurrent neural network (RNN) [17], followed by a feed-forward neural network (FNN). Although these black-box solutions perform better for Implicit relations, they still achieve pretty poor performance for sense classification, train multiple models for each language, and we need to fine-tune their features and embeddings.

In this paper, we move away from hand-engineering and designing a system specifically for a given language. We pursue an end-to-end trainable approach, that is language-independent with respect to its inputs and architecture, and applicable as such to very different languages. Such a system needs to consist of a single model for handling all types and senses of discourse relations (no differences in handling Explicit and Implicit discourse relations). It should not perform any preprocessing of its input text spans, nor require any feature engineering or external resources, not even pre-trained word embeddings. We accomplished all this in our novel *FR system* for sense classification based on our generalization of focused recurrent neural networks (RNNs), that we present in this paper. The *focused RNNs layer* at word level [17] represents the first multi-dimensional RNN-attention mechanism for constructing text/argument embeddings. We improve upon this by processing any sequence of symbols of arbitrary lengths and sharing weights between multiple focused RNNs layers. This way, its filtering/gating RNN enables downstream RNNs to focus on different aspects of the input sequence and project it into several embedding subspaces, called text or argument embeddings. In our model, a FNN is then put on top to perform sense classification. Because of its generic design our model can be easily adapted for other NLP problems in which end-to-end training and multi-dimensional argument embeddings are needed. We successfully applied our model for sense classification on tokenized sentences at the word-level representation of inputs (FR-wa model), but are also the first to present a character-level model for sense classification (FR-ca model). Since we are learning task-specific word embeddings from scratch, we merely apply a simple data augmentation technique during training. To support its language-independence we evaluate it on Chinese, as an example of a less supported language, and on English, as an example of the linguistically most explored and supported language. By following the official task formulation, datasets and methodology of the CoNLL 2016 Shared Task [8], we compare FR system with winning systems and strong baselines on these two languages. FR system does not fall a lot behind state-of-the-art performance on English datasets, and even outperforms existing best systems by 2.5% overall results on the Chinese blind dataset.

We organize the rest of the paper in four main sections. Section Background describes shallow discourse relations and surveys related work. Section Our model describes the FR system architecture, its neural network layers, and our generalization of focused RNNs layer. Section Evaluation presents the official evaluation methodology, and detailed analysis of its performance on Chinese and English datasets. The last section makes a brief overview and draws conclusions.

## Background

Early work on linguistic and computational discourse analysis produced several theoretical frameworks to analyze the language beyond the clause and sentence level [18, 19]. Although they differ in many ways, they all more of less encode problems involving argument extraction [20] and sense classification of Explicit [9] or Implicit discourse relations [21]. Unfortunately, differences in theories, data set creation, features used, label sets, and experimental methodologies make it difficult to compare early works fairly and adequately. For this reason, we adopt the view of lexically-grounded shallow discourse relations, that are annotated in two newswire corpora, the English Penn Discourse TreeBank (PDTB) [19] and the recently published Chinese Discourse Treebank (CDTB) [22]. We perform evaluation on these two datasets according to the CoNLL 2015 and CoNLL 2016 Shared Task [8, 11].

Shallow discourse relations strive to maintain a theory-neutral approach by lexically anchoring relations to discourse connectives, even when they are not explicilty expressed [19]. They occur both across sentences and within sentences, arguments are defined based on the location of the connective, and there are no restrictions on how many clauses and gaps they may contain. The following two examples on Chinese language (also translated to English) show how one sentence contains two overlapping discourse relations and how a connective ($\underline{\underline{\text{conn}}}$) consists of multiple segments.

1. *[*建筑 公司 进 区*]*_*arg1*_ $\underline{,}$ *[*有关 部门 先 送上 这些 法规性 文件, 然后 有 专门 队伍 进行 监督 检查*]*_*arg2*_
  *[Construction companies enter the area]*_*arg1*_ $\underline{,}$ *[relevant departments first send these regulatory documents, and then a special team conducts supervision and inspection]*_*arg2*_.
                                  — Implicit, sense Conditional
2. 建筑 公司 进 区, *[*有关 部门 $\underline{\underline{先}}$ 送上 这些 法规性 文件*]*_*arg1*_ $\underline{,}$ $\underline{\underline{然后}}$ *[* 有 专门 队伍 进行 监督 检查 *]*_*arg2*_
  *Construction companies enter the area, [relevant departments* $\underline{\underline{first}}$ *send these regulatory documents]*_*arg1*_ $\underline{,}$ $\underline{\underline{and\;then}}$ *[a special team conducts supervision and inspection]*_*arg2*_.
                                   — Explicit, sense Temporal

The literature further distinguishes four discourse relation types as can be seen in Table 1, but focuses mostly on Explicit and Implicit discourse relations that occur most often. Each relation type further consists of sense labels, which we try to predict. Due to differences between languages, such as the formalization of the concept of a sentence and the way arguments are labeled, there are 10 sense labels defined for Chinese and 21 for English (see section Evaluation or CoNLL 2016 Shared Task [8] for a complete list). These senses are unevenly distributed, especially in Chinese where more than half of relations signal the sense Conjunction and in English almost a quarter of relations signal Expansion.Conjunction.

**Table 1:** Distribution of relation types in Chinese and English datasets.

Relation type	Chinese datasets				English datasets			
	train	valid	test	blind	train	valid	test	blind
Explicit	2225	77	96	566	14722	680	923	556
Implicit	6706	251	281	1399	13156	522	769	425
AltLex	211	5	7	49	524	19	30	28
EntRel	1098	50	71	87	4133	215	217	200
Total relations	10240	383	455	2101	32535	1436	1939	1209

English dataset contains more than three times as many training examples as Chinese dataset. In English Implicit discourse relations occur almost as often as Explicit ones, but in Chinese as much as twice as often. Each relation type further consists of up to 10 sense labels for Chinese and 21 for English, which we try to predict (EntRel has a dedicated sense label).

A couple of *sense classifiers* have been developed as standalone systems, while others were used as components of a shallow discourse parser. Table 2 presents a comparison of best performing methods for sense classification.

**Table 2:** Comparison of sense classifiers for shallow discourse relations.

	[13]	[14]	[15]	[16]	[23]	[17]	FR system
supported languages	en	en	en, zh	en, zh	en, zh	en, zh	en, zh
supported relation types	All	All	Non-E	All	All	All	All
different sense models	3	2	1	5	3	2	1
external resources	3	2	1	3	2	0	0
hand-engineered features	yes	yes	no	yes	no	no	no
end-to-end trainable	no	no	yes	no	no	no	yes
tokenized input	required	required	required	required	required	required	optional

All previous systems or their components for sense classification consist of substantially different models for English (en) and Chinese (zh) languages and also for handling Explicit, Implicit and other discourse relations. Our FR system at the word (FR-wa) and at the character level (FR-ca) differ in many ways.

Explicit discourse relations use discourse connectives or markers as linguistic expressions, that explicitly signal the presence of a discourse relation between two arguments [24]. It turns out, that using only a list of connectives sets a reasonably high baseline for sense classification in English. Adding more syntactic category features helps to mitigate most ambiguities between their discourse or non-discourse usage [9]. Further improvements can be achieved by also extracting POS tags and features from the context of connectives [13]. The best known approach for English uses a logistic regression classifier with several cross-argument similarity features based on pre-trained word embeddings [14]. For Chinese, the best methods apply SVM on the connectives themselves [23] or apply focused RNNs [17], like we do. Other methods for Chinese Explicit discourse relations use logistic regression classifiers with features similar to English [16], but their performance is slightly worse.

Implicit discourse relations are missing an overt discourse connective and their interpretation needs to rely on semantic meaning and general knowledge about the world. They were first approached with *conventional machine learning* techniques and hand-engineering. Due to the lack of data, early work used patterns to extract explicit discourse examples from unlabeled data, and generated synthetic Implicit discourse relations by just removing the connectives [25]. However, linguistic dissimilarity between explicit and implicit data has to be considered to determine in which cases it is safe to do this [26]. Early supervised approaches relied heavily on hand-engineered lexicons and features derived from syntactic parse trees. Features based on cross-products of words between arguments help, but they are not the semantically-related pairs that researchers hoped for [10]. Another successful approach is massive extraction of production rules, dependency rules, and word pairs followed by a feature selection process [12, 21]. Employing additional features from Brown cluster pairs and coreference patterns improves the results even further [13, 27]. Unfortunately, the crucial role of feature selection and cut-off thresholds indicates that most features are useless and contribute more noise than signal. Despite the use of thousands to millions of features, Naive Bayes with feature selection proved to be the most efficient and consistently best-performing conventional machine learning technique for English Implicit discourse relations [21, 27]. Best conventional methods for Chinese are based on production rules of arguments [28], however some managed to enhance them with word and verb pairs at specific locations to achieve slightly better performance [16].

Methods based on *neural networks* seem to be particularly appealing for processing Implicit discourse relations due to their power of capturing semantic information in their latent vector representations. An early approach computed vector representations of arguments of discourse relations and coreferent entity mentions through a series of compositional operations over the syntactic parse tree [29]. Another approach constructed vector representations of arguments as an average of pre-trained word embeddings combined with Brown clusters [30]. A comparison of one-hot, Brown, and vector representations on word pairs of arguments confirmed that pre-trained word embeddings seem to provide most of the semantic and syntactic information relevant to the task [31]. Best known approaches with neural networks somehow produce vector representations of both arguments and then apply a FNN for classification. The simplest approaches compute only an average of pre-trained word embeddings and achieve state-of-the-art results on English [15], or perform a series of summations and multiplications of pre-trained word embeddings and parse tree depth embeddings [23]. Some apply convolutional neural networks on each argument separately [14, 16], while others use them to produce shared vector representations of cross-argument word pairs in a multi-task environment with different annotation frameworks [32]. State-of-the-art performance on Chinese is achieved by our older two-model system with focused RNNs at word level [17]. It differs from FR system by: using two separate models (one for processing Explicit and one for non-Explicit discourse relations), requires tokenized input at word level, during training only random noise samples are introduced for each discourse relation sample, and it uses many fine-tuned parameters for each model and language. We build upon this approach, because we want our FR system to be easily trainable, handle different label sets and languages, and not depend on external resources.

*Neural attention mechanisms* allow a model to automatically search for the parts of the input that are relevant for processing at each step and adjust its focal point over time. This corresponds with the view, that clauses and sentences are sequences of words where all words do not equally contribute to their meaning and can be perceived with emphasis on different aspects. Initially they were applied in encoder-decoder frameworks, such as image caption generation where a CNN encodes the image and an attention mechanism helps the RNN decode better descriptions [33]. For machine translation, the same concept, but with bidirectional RNN for encoding, successfully learned to align words and translate between English and French [34]. Adding the attention mechanism to a three-layer LSTM model enabled it to successfully perform linearized syntactic constituency parsing [35]. Attention mechanism is also suitable for question answering tasks [36], because it gives the model at each answer generating step a fuzzy access to its internal memory as a weighted average representation of all memory locations. End-to-end memory networks [37] present a different approach by stacking multiple attention layers and updating the question representation at each step.

The core component of FR system is our novel generalization of the *focused RNNs layer*, a neural attention mechanism. The concept of *focused RNNs layer* at word level was introduced by Weiss and Bajec (2016) [17]. It differs greatly from other attention mechanisms and represents the first multi-dimensional RNN-attention mechanism. In contrast to previous mechanisms, all attention weights are computed only once by a filtering RNN and not recomputed at each processing step to focus on a different aspect. Instead of computing a single attention weight for each word, the multi-dimensional approach represents a natural progression of this idea by computing multiple attention weights for each aspect of each word in parallel. Instead of using a primitive weighted average to compute sentence/argument embeddings it applies downstream RNNs to compose information on different aspects of input sequences into argument embedding subspaces. These argument embeddings can later be used for different NLP tasks, such as sense classification. Our generalization of focused RNNs layer further improves upon this by: processing any sequence of symbols of arbitrary lengths (such as character level inputs), sharing weights between multiple focused RNNs layers, using a bidirectional LSTM for filtering RNN, and LSTMs for focused downstream RNNs.

## FR system

In this section, we start with introducing our FR system and its neural network architecture, then provide details for each of the neural network layers it consists of, and finally describe the training process.

**Fig 1:**
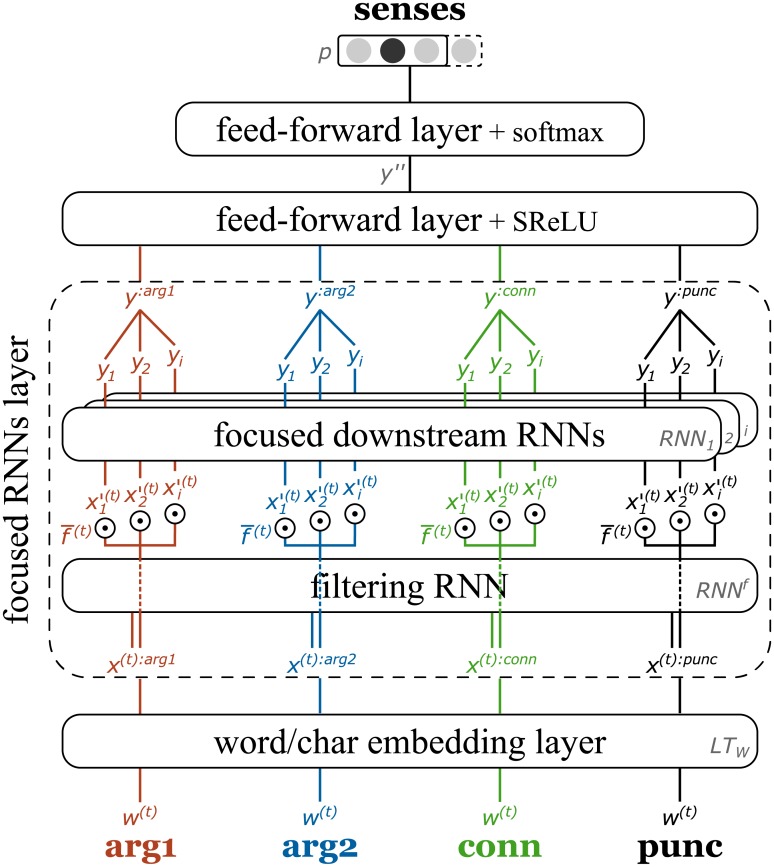
FR system with focused RNNs layer for sense classification. Each of the four text spans are first mapped to word embeddings, then separately processed by our focused RNNs layer to produce multi-dimensional argument embeddings. These are used in a two-layer FNN to perform sense classification.

*FR system* is our proposed solution/method for sense classification of shallow discourse relations. It consists of a single end-to-end trainable neural network, input preparation at word or character levels, and the training procedure with a simple data augmentation technique. The architecture of our neural network with focused RNNs layer is presented in Fig 1. It directly follows the task definition, is designed to be language-independent, can handle all types and senses of discourse relations, requires no feature engineering or external resources, and can be applied at the word level (on tokenized sentences) and even at the character level. The whole model is end-to-end differentiable and can be trained with backpropagation from labeled samples.

The input for each discourse relation is provided in the form of four text spans: for two arguments (arg1, arg2), an optional connective ($\underline{\underline{\text{conn}}}$), and optional punctuation in Chinese (punc). In the spirit of end-to-end training we perform no preprocessing and work directly with concatenated sequences of input symbols at either word or character level (*w*^(0)^, *w*^(1)^, …, *w*^(*t*)^). Without using any pre-trained word embeddings or other resources, in the first layer the model learns to transform input symbols into task-specific vector representations, called word or character embeddings (*x*^(*t*)^). The focused RNNs layer consists of a filtering RNN, a multiplicative filtering/gating mechanism, and several focused downstream RNNs. By multiplying attention weights with the input sequence ($x_{1}^{\prime (t)}$, $x_{2}^{\prime (t)}$, …, $x_{i}^{\prime (t)}$) downstream RNNs can specialize or focus on different aspects of each text span and produce fixed-size vector representations or argument embeddings of different aspects (*y*_1_, *y*_2_, …, *y*_*i*_). All concatenated argument embeddings (*y*^:*arg*1^, *y*^:*arg*2^, *y*^:*conn*^, *y*^:*punc*^) are passed into a two-layer FNN to classify the sense label of a discourse relation (*p*).

We successfully applied the same neural network architecture on Chinese and English datasets at both the word level (FR-wa) and character levels (FR-ca). Due to the differences between languages and different sense labels, a few basic parameters had to be adjusted (see Table 3). In Evaluation, we present detailed evaluation results of each model.

**Table 3:** Adjust basic model parameters for each language.

Parameter	Chinese			English		
	FR-wa	FR-ca	simple	FR-wa	FR-ca	simple
Argument 1 length	500	900	500	100	400	100
Argument 2 length	500	900	500	100	400	100
Connective length	10	20	10	10	20	10
Punctuation length	2	2	2	0	0	0
Focused RNNs	12	12	−	8	8	−
- recurrent dim.	20	20	240	20	20	160
Trainable weights	416,400	177,880	1,309,472	947,119	70,499	1,236,943
- word emb. (20-dim)	295,700	57,180	295,700	878,360	1,740	878,360
- other layers	120,700	120,700	1,013,772	68,759	68,759	358,583

Due to the differences between languages and different sense labels, we need to adjust the maximal lengths of text spans and the number of focused downstream RNNs. Our model at the word (FR-wa) and at the character level (FR-ca) have far fewer trainable weights than the baseline model with simple LSTMs (simple).

### Word or character embeddings layer

The first layer of our model transforms input symbols at either word or character level (*w*^(*t*)^) into task-specific vector representations (*x*^(*t*)^) suitable for neural networks, called word embeddings [38] or character embeddings. We train these embeddings from scratch and do not use any other resources provided by the CoNLL 2016 Shared Task [8], such as POS tags, syntactic parse trees, dependency parses, Brown clusters, or pre-trained word embeddings.

If we process input at the word level (as in *FR-wa model*), we use the fact that all datasets provide each discourse relation in the form of four text spans already tokenized/segmented into words and punctuation. We represent them as four concatenated sequences of words or tokens (*w*^(*t*)^). Due to technical limitations, we crop longer sequences depending on the language (see Table 3). Initially our method scans the whole training dataset to build a vocabulary of known words. A special out-of-vocabulary symbol is reserved for unseen words, that may be encountered later.

If we process input at the character level (as in *FR-ca model*), we represent each discourse relation as four concatenated sequences of characters (*w*^(*t*)^), including white-spaces, punctuation, and other symbols. Because there are far fewer different characters as there are words and more characters per sentence than words, maximal lengths of sequences are longer (see Table 3). Initially our method scans the whole training dataset to build a vocabulary of known characters. A special out-of-vocabulary symbol is reserved for the improbable event, that unseen characters are encountered later. There are several benefits of using character-level representations over word-level: they do not suffer from out-of-vocabulary issues, are able to model different and rare morphological variants of a word, and do not require tokenization/segmentation.

The *word or character embeddings layer* are computed in the same way. It can be thought of as a lookup table *LT*_*W*_(⋅), that maps each input symbol *w*^(*t*)^ from the vocabulary into a fixed-size vector representation *x*^(*t*)^ of real numbers, called word or character embedding,

$$\begin{array}{c} x^{\left(t\right)}=LT_{W}\left(w^{\left(t\right)}\right) \end{array}$$ (1)

where (*t*) is the time dimension in the sequence, and *W* a trainable matrix for the lookup table.

Although a couple of pre-trained word embedding lookup tables exist for different languages, such as Word2vec [39] or GloVe [40], we achieved better results by learning task-specific word embeddings from scratch. On the other hand, there are no pre-trained character embeddings, so we also had to learn task-specific character embeddings from scratch. These embeddings automatically emerge when training the whole model in an end-to-end manner using backpropagation. Even though some experiments suggest that optimal word embeddings are dependent on discourse relations [31], the lack of large amounts of training data makes it unrealistic to learn separate word embeddings. By applying the same word or character embedding layer to all four text spans, all input symbols are thus represented in the same vector space.

### Focused RNNs layer

The *focused RNNs layer* can analyze different aspects of input sequences of word embeddings (*x*^(*t*)^) and project them into fixed-size vector representations, called sentence or argument embeddings (*y*). Our work builds upon the concept of focused RNNs layer at word level [17].

**Fig 2:**
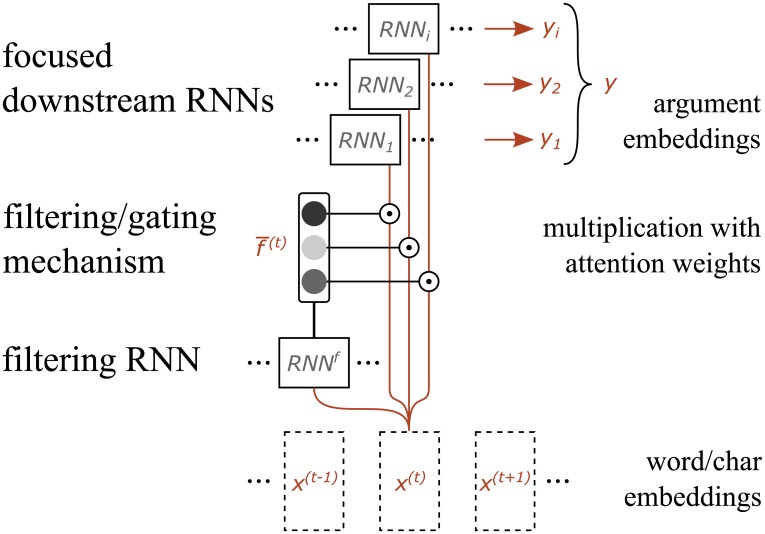
Focused RNNs layer consists of a filtering RNN, a filtering mechanism, and focused downstream RNNs. For each word embedding the filtering RNN produces a vector of attention weights. The filtering mechanism multiplies each weight with the same word embedding to produce a weighted word embedding. Each focused downstream RNNs then projects these into an argument embedding subspace.

Each text span represented as a sequence of word embeddings (*x*^(*t*):*arg*1^, *x*^(*t*):*arg*2^, *x*^(*t*):*conn*^, *x*^(*t*):*punc*^) is processed by a separate focused RNNs layer as presented in Fig 2. For each word embedding the filtering RNN (*RNN*^*f*^) first produces a vector of attention weights (${\bar{f}}^{\left(t\right)}$). The filtering/gating mechanism multiplies each weight with the same word embedding to produce a weighted word embedding ($x_{i}^{\prime (t)}$). The weighted input sequence produced in this way makes it possible for downstream RNNs (*RNN*_*i*_) to focus on different aspects and project each one into a fixed-size vector representation (*y*_*i*_) in an argument embedding subspace. Afterwards these vectors are separately concatenated for each text span to produce four argument embeddings (*y*^:*arg*1^, *y*^:*arg*2^, *y*^:*conn*^, and *y*^:*punc*^).

The intuition behind it is that different downstream RNNs can specialize or focus on different aspects of each text span in parallel and independently of one another. Due to the black-box nature of neural networks it is unclear what these aspects represent. It seems that the optimal number of focused RNNs depends on the language and task (see Table 3), but not on sense labels or their distribution. In its internal state each focused RNN composes a fixed-size vector representation in an argument embedding subspace, that can be used in subsequent layers to solve a given task. To the best of our knowledge, this represents the first multi-dimensional RNN-attention mechanism. By applying the same focused RNNs layer to each text span, we encourage them to compose information on different aspects of input sequences into the same argument embedding space, instead of overfitting on specifics of each text span.

First the *filtering RNN* (*RNN*^*f*^) produces a vector of attention weights *f*^(*t*)^ between [0, 1] for each position (*t*) of the input sequence. These can be interpreted as the relative importances of position (*t*) during blending of different aspects of the input sequence. Theoretically any type of RNN can be used for the filtering RNN, but a bidirectional LSTM layer [41] with the *σ* activation function performs somewhat better, because it can capture long-term dependencies from preceding and succeeding input symbols. LSTM [42] is a commonly used RNN that features an internal memory cell *c*^(*t*)^ whose manipulation and usage is controlled with an input *g*^*i*^, forget *g*^*f*^, and output *g*^*o*^ gates. It can be described with

$$\begin{array}{c} \begin{array}{cc} g^{i} & =\sigma (W^{i}x^{\left(t\right)}+U^{i}f^{\left(t-1\right)})\\ g^{f} & =\sigma (W^{f}x^{\left(t\right)}+U^{f}f^{\left(t-1\right)})\\ g^{o} & =\sigma (W^{o}x^{\left(t\right)}+U^{o}f^{\left(t-1\right)})\\ c^{\left(t\right)} & =g^{f}⊙c^{\left(t-1\right)}+g^{i}⊙\sigma \left(W^{c}x^{\left(t\right)}+U^{c}f^{\left(t-1\right)}\right)\\ f^{\left(t\right)} & =g^{o}⊙\sigma \left(c^{\left(t\right)}\right) \end{array} \end{array}$$ (2)

where ⊙ represents element-wise multiplication (Hadamard product). *W*^*i*^, *W*^*f*^, *W*^*o*^, *U*^*i*^, *U*^*f*^, *U*^*o*^ are trainable matrices, and *c*^(−1)^ and *f*^(−1)^ are the initial hidden states. The bidirectional LSTM has two sets of these formulas differing only in the direction of processing the time dimension. The output vectors *f*^(*t*)^ at matching positions from both directions are then averaged to produce the attention weights ${\bar{f}}^{\left(t\right)}$. We should share the weights of all filtering RNN globally to ensure that the attention weights for each text span are produced by the same mechanism.

Afterwards we apply a multiplicative *filtering/gating mechanism* to regulate how much of the input signal should be passed to different focused downstream RNNs. For each position (*t*) of the input sequence we have an input vector *x*^(*t*)^, usually a word embedding, and a vector of attention weights ${\bar{f}}^{\left(t\right)}$ from the filtering RNN. We separately multiply the input vector with each dimension of attention weights ${\bar{f}}_{\left[i\right]}^{\left(t\right)}$ in order to direct the attention to different aspects of the input sequence

$$\begin{array}{c} \begin{array}{cc} \left[{\bar{f}}^{\left(0\right)},{\bar{f}}^{\left(1\right)},\ldots ,{\bar{f}}^{\left(t\right)}\right] & =RNN^{f}\left(\left[x^{\left(0\right)},x^{\left(1\right)},\ldots ,x^{\left(t\right)}\right]\right)\\ x_{i}^{\prime (t)} & ={\bar{f}}_{\left[i\right]}^{\left(t\right)}x^{\left(t\right)} \end{array} \end{array}$$ (3)

where the result of *RNN*^*f*^(⋅) is a sequence of averaged output vectors for each position from the filtering RNN. $x_{i}^{\left(t\right)}$ represents the weighted vector to be passed to the *i*-th focused downstream RNN (*RNN*_*i*_).

Each *focused downstream RNN* (*RNN*_*i*_) receives a sequence of weighted inputs $\left[x_{i}^{\prime (0)},x_{i}^{\prime (1)},\ldots ,x_{i}^{\prime (t)}\right]$ and composes a fixed-size vector representation of its aspect *y*_*i*_ in its argument embedding subspace. For usage in classification tasks, such as sense classification, the vectors of all aspects are then concatenated to form the final argument embedding (eg. *y*^:*arg*1^ for arg1)

$$\begin{array}{c} \begin{array}{cc} y_{i} & =RNN_{i}\left(\left[x_{i}^{\prime (0)},x_{i}^{\prime (1)},\ldots ,x_{i}^{\prime (t)}\right]\right)\\ y^{:arg1} & =Concat(\left[y_{1},y_{2},\ldots ,y_{i}\right]) \end{array} \end{array}$$ (4)

where the result of *RNN*_*i*_(⋅) is the last internal state of the *i*-th focused RNN, and *Concat*(⋅) represents concatenation of vectors. Any type of RNN can be used for focused RNNs (*RNN*_*i*_), but we did not observe any substantial gains in using specific RNNs, probably due to already weighted inputs from the filtering mechanism. To avoid introducing new methods, we use same forward-only LSTM for focused downstream RNNs. We should apply the same set of focused downstream RNNs (*RNN*_*i*_) to each input sequence. This encourages each downstream RNN to specialize or focus on a different aspect and project each aspect into the same argument embedding subspace, instead of overfitting on specifics of each input sequence.

Note that the concept of focused RNNs layer differs greatly from other neural attention mechanisms. Differences are described in section Background.

### Feed-forward classification layer

Finally, all concatenated argument embeddings (*y*^:*arg*1^, *y*^:*arg*2^, *y*^:*conn*^, *y*^:*punc*^) are passed into a two-layer *feed-forward neural network* (FNN) to perform classification of the sense label (*p*).

First all argument embeddings are processed by a feed-forward layer with the SReLU activation function, afterwards another feed-forward layer with the Softmax activation function is put on top to compute the classification probability distribution *p*

$$\begin{array}{c} \begin{array}{cc} y^{\prime } & =Concat(\left[y^{:arg1},y^{:arg2},y^{:conn},y^{:punc}\right])\\ y^{\prime \prime } & =SReLU(W^{\prime }y^{\prime }+b^{\prime })\\ p & =Softmax(W^{\prime \prime }y^{\prime \prime }+b^{\prime \prime }) \end{array} \end{array}$$ (5)

where *Concat*(⋅) represents concatenation, and *SReLU*(⋅) and *Softmax*(⋅) represent the corresponding activation functions, whose details are described below.

The S-shaped rectified linear activation unit (SReLU) [43] consists of a piecewise linear function with three parts. It is defined as

$$\begin{array}{c} SReLU\left(z_{i}\right)=\{\begin{array}{cc} t_{i}^{l}+a_{i}^{l}\left(z_{i}-t_{i}^{l}\right) & z_{i}\leq t_{i}^{l}\\ z_{i} & t_{i}^{l}<z_{i}<t_{i}^{r}\\ t_{i}^{r}+a_{i}^{r}\left(z_{i}-t_{i}^{r}\right) & t_{i}^{r}\leq z_{i} \end{array} \end{array}$$ (6)

where $t_{i}^{r},a_{i}^{r},t_{i}^{l},a_{i}^{l}$ are four trainable parameters and the subscript *i* indicates that we allow SReLU to vary in different dimensions of its input vectors. Due to its construction it is capable of learning both convex and non-convex functions. This makes the SReLU activation function perform somewhat better for sense classification than the convex-only ReLU activation or without an activation function, but is faster to compute than traditional trigonometric functions.

The Softmax activation function computes a probability vector over its inputs, like a logistic regression. It is defined as

$$\begin{array}{c} Softmax\left(z_{i}\right)=\frac{e^{z_{i}}}{\sum_{j}e^{z_{j}}} \end{array}$$ (7)

### Training and implementation

Training can be performed on the training dataset with backpropagation with any gradient descent optimization algorithm. We chose to use the Adam optimizer [44] because it is well suited for problems that incorporate many parameters. To parallelize and speed up the learning process, training is done in mini-batches of 64 training samples. We use masking to process variable sizes of text spans and use fixed mini-batch dimensions. In addition to tracking the training loss and validation loss functions, we also periodically evaluate the performance of our model using the official evaluation methodology of CoNLL 2016 Shared Task. The training procedure is stopped when there is no improvement on the validation dataset in last 10 real epochs.

A suitable loss function or training objective for sense classification is the categorical cross-entropy error function, also known as multi-class log-loss. The goal is to minimize the difference between the computed approximating distribution *p* and the one-hot vector encoding of the true sense labels provided on the training data.

FR system for sense classification is available at http://github.com/gw0/conll16st-v35-focused-rnns/ under the AGPL-3.0+ license and implemented in Python 2.7 using the Keras 1.2.2 library [45]. The Keras library provides a high-level API for developing neural networks, it is based on Theano and Tensorflow, and is capable of running on either CPU or GPU. All models and their training procedures are implemented in Keras.

It is impossible to compare the computational time required by FR system with other systems, because others did not report this information. Training FR system at word level on the English dataset takes around 8 hours on a PC with a middle-range GPU, while performing predictions on the English test dataset takes around 48 seconds. Its prediction times are among the fastest if compared to submissions of CoNLL 2016 Shared Task.

#### Data augmentation

During training we perform a simple data augmentation technique that makes the model more robust to noise and improves the learning of task-specific word or character embeddings from scratch. We transform each original discourse relation in the training dataset into 2 positive and 2 negative samples.

For positive samples the sense label remains the same, because we introduce only so little noise, that it should not affect the overall meaning. This improves the robustness of the classifier with respect to noise in data. For positive samples there is a 30% probability that 10% of symbols in arg1 and arg2 get mutated by each of the following three functions:

- duplicate a randomly chosen symbol (it may may be important)
- insert at random the out-of-vocabulary symbol (it may -OOV- be important)
- forget a randomly chosen symbol (it -OOV- be important)

For negative samples we use a special no-sense label, because at least a part of them is always replaced with random symbols from the vocabulary, thus the text itself does not make any sense and there is no discourse relation anymore. This also improves the robustness of intermediate representations and counteracts the need to normalize word or character embeddings on the whole vocabulary. For negative samples first $\underline{\underline{\text{conn}}}$ and punc are always replaced with random symbols, if present. Afterwards there is a 70% probability that arg1 and arg2 get mutated by each of the following three functions (and in the unlikely event, that nothing changed, everything is replaced with random symbols of maximal length):

- replace arg1 with random symbols of same length (admission Charles crash $ going Witter 20.9 daily Kidder million is -- ignoring cloud & list)
- replace arg2 with random symbols of same length
- swap arg1 and arg2

#### Hyper-parameters

We use the same model hyper-parameters on all setups to make it usable almost out-of-the-box on any language, label set, and level of input representation. It is interesting to note, that attempts at fine-tuning the model hyper-parameters for different setups did not substantially improve its performance. Due to the differences in average sentence and token lengths, and number of sense labels, a few basic parameters had to be adjusted as described in Table 3. Maximal lengths of text spans were determined in the way that 99% of discourse relations are not modified. The optimal number of focused RNNs depends on the language and not on whether it is applied at word or character level. All other parameters should use the values described in this subsection.

It is sufficient that the dimensionality of the word embedding layer is only 20, of the filtering RNN layer 8 for English and 12 for Chinese (to match the number of focused RNNs), of focused downstream RNNs 20, and of the FNN hidden layer 80. Our goal is to predict only sense labels, there are 11 for Chinese and 22 for English, including partially annotated senses and a special no-sense label.

Initial values of trainable weights are set according to best practices, as they do not affect the training outcome substantially. The word embedding layer is therefore initialized with a uniform random distribution, all RNN layers with Glorot uniform random distribution [46] and inner cells with an orthogonal matrix, and all FNN layers and the slope of the SReLU activation function again with Glorot uniform random distribution.

Due to many trainable parameters and the lack of training samples, we improve the generalizability of our model with dropout layers [47] and weight sharing. Dropout is a well-known regularization technique that reduces overfitting in neural networks by preventing complex co-adaptations in the training dataset. We introduce dropout layers with 0.3 fraction of entries that will be randomly set to 0 at each update during training time. We add them after each major layer of our model: after the word embedding layer, after the concatenated argument embeddings of focused RNNs, and after the FNN hidden layer before classification. We also performed some experiments with dropout and zoneout regularization on recurrent connections of RNNs, but there were no substantial improvements. Furthermore, we tried to introduce curriculum learning by gradually increasing the length of arguments during training but again, with no substantial improvements. To improve the generalizability, our model also performs sharing of trainable weights for word embedding, filtering RNN, and focused RNN layers, as described in previous subsections. Experiments have shown that disabling the weight sharing degrades the performance of our model for sense classification.

## Evaluation

To support that FR system is language-independent with respect to its inputs and architecture we apply it as such on Chinese, as an example of a less supported language, and on English, as the language with most research and advanced language technologies. We follow the official task formulation, datasets and evaluation methodology of the CoNLL 2016 Shared Task [8], organized within the conference. The competition focused on shallow discourse parsing and sense classification on these two languages.

Performance is computed using the *F*_1_-score based on the number of relations that match a gold-standard relation exactly by comparing only the sense labels. In cases where the gold-standard relation is annotated with two senses, the predicted sense must match one of these senses to be considered correct. In cases where the gold-standard sense label is only partially annotated, the predicted sense must match the partially annotated sense (although the blind datasets do not contain partial annotation). Official ranking is based on the overall performance on blind test datasets.

### Chinese datasets

The Chinese datasets for the CoNLL 2016 Shared Task were adapted from the Chinese Discourse Treebank 0.5 (CDTB 0.5) [22] and the Chinese Wikinews. The CDTB 0.5 follows the general annotation strategy of the PDTB 2.0 but adapts it to the Chinese language. The training dataset contains 10240 relations from CDTB 0.5, the validation dataset contains 383, and the testing dataset 455. The official ranking is based on the slightly out-of-domain blind test dataset to evaluate robustness, which contains 2101 relations from 64 articles from the Chinese Wikinews. Table 1 shows the distribution of discourse relations in these datasets according to relation types that are not directly relevant to sense classification. Most frequent are Implicit discourse relations that occur in Chinese three times as often as Explicit ones. These datasets also contain POS tags, syntactic parse trees, and dependency parses, but in our approach we ignore this information.

Since the concept of a sentence is less formalized in Chinese, arguments of discourse relations in Chinese are less evenly distributed as in English and defined semantically. Consequently, we predict a flat set of 10 sense labels. Inter-annotator agreement on sense labels on the CDTB 0.5 is overall 87.4% with expected agreement for Explicit relations better than for non-Explicit types of relations.

### Results for Chinese

In Table 4 we compare our results for sense classification on the Chinese datasets with winning systems of the CoNLL 2016 Shared Task [8] and strong baselines:

- Weiss and Bajec (2016) [17] is our older two-model system, that was the overall top performing system of the CoNLL 2016 Shared Task. It uses two separate models with focused RNNs at word level, many fine-tuned parameters, and trains with random noise samples, but uses no external resources.
- Schenk et al. (2016) [23] uses a SVM classifier on the connectives themselves for Explicit discourse relations, and for other a series of summations and multiplications of word and parse tree depth embeddings. It uses pre-trained word embeddings and parse trees.
- Wang and Lang (2016) [16] uses logistic regression classifiers with many hand-engineered features from the connective and its context for Explicit discourse relations, and production rules and features with word and verb pairs at specific locations for non-Explicit. It uses POS tags, parse trees, and word categories.
- most common class is a minimal baseline with no predictive power (uses Conjunction).
- simple LSTMs + augm. is a strong baseline model with 240-dimensional LSTM layers instead of the focused RNNs layer in our system. Due to it having more than three times as many trainable weights than our model, it should be far more powerful. It trains with our data augmentation, but uses no external resources.

**Table 4:** Overall results on Chinese datasets.

Models	valid			test			blind		
	All	Exp	Non-E	All	Exp	Non-E	All	Exp	Non-E
**FR system**									
- word level	0.7285	0.9481	0.6743	0.7437	0.9319	0.6936	0.7120	0.7951	0.6814
- word level + augm. (FR-wa)	0.7520	0.9351	0.7070	0.7363	0.9375	0.6825	0.7396	0.7597	**0.7322**
- word level + word2vec + augm.	0.7493	0.9481	0.6993	0.7297	0.9479	0.6713	0.7373	0.7827	0.7205
- char level	0.7180	0.8961	0.6743	0.7253	0.9271	0.6713	0.7454	0.7862	0.7303
- char level + augm. (FR-ca)	0.7415	0.9351	0.6939	0.7253	0.9271	0.6713	**0.7477**	**0.8463**	0.7114
**Prior work**									
- Weiss and Bajec (2016) [17]	0.7206	0.9351	0.6667	0.7011	0.9271	0.6407	0.7292	0.7898	0.7068
- Schenk et al. (2016) [23]	0.7572	0.9610	0.7059	0.7701	0.9634	0.7187	0.6373	0.8039	0.5759
- Wang and Lang (2016) [16]	0.7807	0.9610	0.7353	0.7701	0.9424	0.7242	0.6473	0.7669	0.6052
**Baseline models**									
- most common class	0.5770	0.4156	0.6176	0.6110	0.5208	0.6351	0.5788	0.2880	0.6860
- simple LSTMs + augm.	0.7363	0.9221	0.6895	0.7231	0.8854	0.6797	0.6921	0.7968	0.6534

Overall results for all, Explicit and non-Explicit discourse relations on Chinese datasets expressed in *F*_1_-scores using the CoNLL 2016 Shared Task methodology. Our FR system at word (FR-wa) and character levels (FR-ca) with data augmentation outperform all other systems on the blind dataset.

FR system at word (FR-wa) and character levels (FR-ca) with data augmentation improves on overall state-of-the-art performance by 2.5% on the Chinese blind dataset (see Table 4). In comparison to systems not using focused RNNs, it improves by more than 8%, despite using only a single end-to-end trainable model, no hand-engineered features, or external resources. For Explicit discourse relations our models perform comparably to Schenk et al. (2016) [23], which is much simpler. Having a simpler model might be beneficial because there are far fewer Explicit training samples available in Chinese datasets. For non-Explicit discourse relations it is interesting to note, that the performance of most approaches on the Chinese blind dataset is far below the most common class baseline. This clearly suggests that Schenk et al. (2016) [23] and Wang and Lang (2016) [16] overfit the training domain and style of the CDTB 0.5 datasets. Our models at both word (FR-wa) and character level (FR-ca) capture the target concepts better, improving even on our older two-model by Weiss and Bajec (2016) [17].

Additionally, we perform an ablation study to qualitatively assess the contribution of our simple data augmentation technique (+ augm.) and the introduction of pre-trained word embeddings (+ word2vec). These 300-dimensional pre-trained word embeddings were produced by the Skip-gram model from Word2vec [39] on the Gigaword simplified Chinese dataset. The results indicate that introducing data augmentation mostly improves the performance at word level for non-Explicit discourse relations and at character level for Explicit discourse relations. Contrary to expectations, introducing pre-trained word embeddings does not seem to substantially improve the performance. This suggests that the same semantic and syntactic information relevant for sense classification can also be learned from scratch.

Previous studies [26] suggest that there is a substantial difference between Explicit and non-Explicit discourse relations, thus we continue with a detailed analysis of Chinese results in both situations. Because the Chinese datasets are small and discourse relations are less evenly distributed (see Table 1), many sense labels only have a few training samples. Removing them from target classes for classification would probably improve the overall performance, but we reject this idea because it is in conflict with our goal of automatically training our model in an end-to-end manner.

Table 5 shows per-sense results for Explicit discourse relations on Chinese datasets. As expected, the results on the validation and test datasets are better because they originate from the same CDTB 0.5 corpus as the training dataset. On the slightly out-of-domain blind test dataset we see a degradation of more than 10% for two most common sense labels, Conjunction and Expansion. This suggests that they are realized differently in the blind dataset, and manual feature engineering, which disambiguates their meaning could substantially improve the results. Nevertheless, we see that our model at character level (FR-ca) achieves the best results with a large margin for most common sense labels, especially Conjunction, Contrast and Purpose. On the other hand, our model at word level (FR-wa) still achieves competitive overall performance. The strength of incorporating linguistic knowledge and hand-engineered features into a system, as in Wang and Lang (2016) [16], is reflected in better performance for sense labels with only a few samples, such as Alternative and Progression.

**Table 5:** Per-sense results for Explicit discourse relations on Chinese datasets.

Sense	valid		test		blind					
	FR-wa	FR-ca	FR-wa	FR-ca	FR-wa	FR-ca	Weiss	Schenk	Wang	simple
Alternative	−	−	−	−	0.	0.	0.	0.0952	**0.1000**	0.
Causation	1.0000	1.0000	1.0000	1.0000	0.8850	**0.9524**	0.9434	**0.9524**	0.9216	0.9434
Conditional	1.0000	0.9091	0.8000	0.8000	0.7294	0.8077	0.8454	0.8866	0.8791	**0.9231**
Conjunction	0.9275	0.9412	0.9615	0.9495	0.7930	**0.8515**	0.7726	0.7711	0.7324	0.7673
Contrast	0.8750	0.8750	1.0000	0.8571	0.7482	**0.8452**	0.7564	0.7571	0.7245	0.7639
EntRel	−	−	−	−	−	−	−	−	−	−
Expansion	1.0000	1.0000	0.8000	0.9333	0.5714	0.7500	0.7727	**0.7907**	0.7556	0.7273
Progression	0.	0.	0.	0.	0.	**0.3333**	0.	0.2857	**0.3333**	**0.3333**
Purpose	1.0000	1.0000	1.0000	1.0000	0.6667	**0.9474**	0.8182	0.9000	0.9000	0.8571
Temporal	0.9412	0.9412	0.9091	0.9143	0.8143	0.9259	0.8659	**0.9299**	0.8591	0.8774
**Overall**	0.9351	0.9351	0.9375	0.9271	0.7597	**0.8463**	0.7898	0.8039	0.7669	0.7968

Results for each sense label for Explicit discourse relations on Chinese datasets expressed in *F*_1_-scores using the CoNLL 2016 Shared Task methodology.

Table 6 shows per-sense results for all of the other discourse relation types (Implicit, AltLex, and EntRel) on Chinese datasets. The distribution of sense labels for Chinese non-Explicit discourse relations is highly unbalanced. The most common sense, Conjunction, occurs approximately 5-times more frequently than the second and the third sense. Therefore, the overall results are highly correlated with the performance on sense Conjunction. All systems based on focused RNNs perform much better on the sense Conjunction and therefore substantially outperform other systems. Our model at word level (FR-wa) also seems to better capture the target concepts of Contrast and Expansion, which makes it the best overall performing system. Although the training dataset contains many samples of the EntRel discourse relations, our approach seems incapable of automatically learning the concept of coreferent entity mentions. On the other hand, the hand-engineered system by Wang and Lang (2016) [16] outperforms on two very low-frequent senses, Alternative and Progression, but fails on most more-frequent ones, especially Causation and Conjunction. Overall, the performance of our systems improves on state-of-the-art, despite using only a single end-to-end trainable model, no hand-engineered features or external resources, and substantially outperforms other systems not based on focused RNNs.

**Table 6:** Per-sense results for non-Explicit discourse relations on Chinese datasets.

Sense	valid		test		blind					
	FR-wa	FR-ca	FR-wa	FR-ca	FR-wa	FR-ca	Weiss	Schenk	Wang	simple
Alternative	−	−	−	−	0.	0.	0.	**0.5000**	**0.5000**	0.
Causation	0.2667	0.1538	0.2500	0.3529	0.2333	**0.2712**	0.1754	0.0392	0.0755	0.1481
Conditional	0.	1.0000	0.	0.	0.	0.	0.	0.	0.	0.
Conjunction	0.8198	0.8035	0.8068	0.8022	**0.8388**	0.8278	0.8213	0.7294	0.7442	0.7843
Contrast	0.	0.	0.	0.	0.1200	0.0800	0.0784	**0.1481**	0.0408	0.0816
EntRel	0.3714	0.0392	0.3301	0.	0.1449	0.	0.	0.1982	**0.2090**	0.1926
Expansion	0.4800	0.5926	0.4000	0.4516	**0.5455**	0.3825	0.5250	0.4387	0.5024	0.4171
Progression	−	−	−	−	0.	0.	0.	0.2857	**0.3333**	0.
Purpose	0.6667	0.6667	0.	0.	0.1333	0.0690	**0.3333**	0.1250	0.1250	0.2857
Temporal	−	−	0.6667	1.0000	0.3333	0.4211	0.3333	0.3636	0.3000	**0.4800**
**Overall**	0.7070	0.6825	0.6797	0.6713	**0.7322**	0.7114	0.7068	0.5759	0.6052	0.6534

Results for each sense label for Implicit, AltLex, and EntRel discourse relations on Chinese datasets expressed in *F*_1_-scores using the CoNLL 2016 Shared Task methodology.

### English datasets

The English datasets for the CoNLL 2016 Shared Task were adapted from the Penn Discourse TreeBank 2.0 (PDTB 2.0) [19] and the English Wikinews. The PDTB 2.0 annotates shallow discourse relations over the one million word corpus from Wall Street Journal. The training dataset contains 32535 relations from Sections 2-21 of the PDTB 2.0, the validation dataset contains 1436 relations from Section 22, and the testing dataset contains 1939 relations from Section 23. The official ranking is based on the slightly out-of-domain blind test dataset to evaluate robustness, which contains 1209 relations from 71 articles from the English Wikinews. Table 1 shows the distribution of discourse relations in these datasets according to relation types, that are not directly relevant to sense classification. Most frequent are Explicit discourse relations that occur in English almost as often as Implicit ones. These datasets also contain POS tags, syntactic parse trees, and dependency parses, but in our approach we ignore this information.

Sense labels in all English datasets are organized in a three-level hierarchy adopted from the PDTB 2.0. To reduce some sparsity without losing too much of the semantics, some senses from the original PDTB 2.0 annotation have been merged. As a result there are only 21 different sense labels, including partially annotated senses, that we need to predict. Inter-annotator agreement on senses on the blind test dataset is overall 85.5% with better agreement for Explicit relations than for non-Explicit types of relations.

### Results for English

In Table 7 we compare our results for sense classification on the English datasets with winning systems of the CoNLL 2016 Shared Task [8] and strong baselines:

- Mihaylov and Frank (2016) [14] is the overall top performing system of the CoNLL 2016 Shared Task. It uses a predefined list of discourse connectives and two logistic regression classifiers on argument embeddings and several cross-argument similarity features based on pre-trained word embeddings and POS tags.
- Rutherford and Xue (2016) [15] uses a pooling function on word embeddings of each argument followed by a three-layer FNN. It uses pre-trained word embeddings, and is specialized for non-Explicit discourse relations.
- most common class is a minimal baseline with no predictive power (uses Expansion.Conjunction).
- simple LSTMs + augm. is a strong baseline model with 160-dimensional LSTM layers instead of the focused RNNs layer in our system. Due to it having one third more trainable weights than our model, it should be far more powerful. It trains with our data augmentation, but uses no external resources.

**Table 7:** Overall results on English datasets.

Models	valid			test			blind		
	All	Exp	Non-E	All	Exp	Non-E	All	Exp	Non-E
**Focused RNNs**									
- word level	0.5788	0.8823	0.3124	0.5618	0.8764	0.2754	0.5062	0.7561	0.2940
- word level + augm. (FR-wa)	0.5930	0.8756	0.3447	0.5416	0.8460	0.2646	0.5170	0.7230	0.3415
- word level + word2vec + augm.	0.6009	0.9029	0.3351	0.5886	0.8970	0.3080	0.5182	0.7676	0.3063
- char level	0.5845	0.8968	0.3102	0.5385	0.8753	0.2321	0.5072	0.7237	0.3246
- char level + augm. (FR-ca)	0.5987	0.8907	0.3420	0.5442	0.8753	0.2430	0.5041	0.7279	0.3139
**Prior work**									
- Mihaylov and Frank (2016) [14]	0.6413	0.9120	0.4032	0.6331	0.8980	0.3919	**0.5460**	**0.7820**	0.3451
- Rutherford and Xue (2016) [15]	−	−	0.4032	−	−	0.3613	−	−	**0.3767**
**Baseline models**									
- most common class	0.2202	0.2807	0.1669	0.2088	0.2701	0.1530	0.2738	0.3903	0.1746
- simple LSTMs + augm.	0.6229	0.9120	0.3685	0.5643	0.8796	0.2774	0.5017	0.7766	0.2680

Overall results for all, Explicit and non-Explicit discourse relations on English datasets expressed in *F*_1_-scores using the CoNLL 2016 Shared Task methodology. Our FR system at word (FR-wa) and character levels (FR-ca) with data augmentation fall behind state-of-the-art performance, but do not use any linguistic knowledge or external resources.

FR system at word (FR-wa) and character levels (FR-ca) with data augmentation do not fall a lot behind state-of-the-art performance on the English blind dataset (see Table 7), despite using only a single end-to-end trainable model, no hand-engineered features, or external resources. As expected, Explicit discourse relations are classified better by Mihaylov and Frank (2016) [14], who use a predefined list of discourse connectives. From the results on non-Explicit discourse relations, we see that their cross-argument similarity features overfit the training domain and style of PDTB 2.0 datasets and perform similar to our approach on the blind dataset. On the other hand, the specialized system by Rutherford and Xue (2016) [15] achieves better results. This suggests that a simpler approach with pre-trained word embeddings can capture the target concept better. Additionally, significantly lower *F*_1_-scores of all competing systems for English than for Chinese indicate, that sense classification on English is much more difficult than on Chinese and *F*_1_-scores are highly affected by differences in grammar, sense labels, and their distribution.

We also perform an ablation study to qualitatively assess the contribution of our simple data augmentation technique (+ augm.) and the introduction of pre-trained word embeddings (+ word2vec). These 300-dimensional pre-trained word embeddings were produced by the Skip-gram model from Word2vec [39] on the Google News English dataset. The results indicate that introducing data augmentation usually improves the performance. Introducing pre-trained word embeddings seems to only improve the performance on Explicit discourse relations. This suggests that the same semantic and syntactic information relevant for Implicit discourse relations can also be learned from scratch.

Previous studies [26] suggest that there is a substantial difference between Explicit and non-Explicit discourse relations, therefore we continue with a detailed analysis of Chinese results in both situations. Basic statistics show that there are substantial differences on the distribution of discourse senses. Some senses, like Expansion.Exception, are only present in the training dataset and no other dataset, while 6 others contain merely a few training samples and are not even present in the blind test dataset. If one would remove these senses from the model, there would be fewer target classes for classification and consequently the overall performance would improve. We reject this idea because it is in conflict with our goal of automatically training our model in an end-to-end manner.

Table 8 shows per-sense results for Explicit discourse relations on English datasets. As expected, the results on the validation and test datasets are better, because they are from the same PDTB 2.0 corpus as the training dataset. Although the training dataset contains many samples for Comparison.Contrast, Expansion.Instantiation and Temporal.Synchrony, we see a degradation of more than 15% in *F*_1_-score for all systems on the slightly out-of-domain blind test dataset. This suggests that they are realized differently in the blind dataset, and manual feature engineering, which disambiguates their meaning, could substantially improve the results. Our method at character level (FR-ca) seems to learn the correct concept of senses Contingency.Cause.Reason, Contingency.Condition, and Expansion.Conjunction slightly better than other methods. It is competitive for Expansion.Conjunction, Temporal.Asynchronous.Precedence, and Temporal.Asynchronous.Succession, but falls behind in performance for other senses. Overall, the performance of our models is not far behind state-of-the-art, despite using only a single end-to-end trainable model, no hand-engineered features, or external resources.

**Table 8:** Per-sense results for Explicit discourse relations on English datasets.

Sense	valid		test		blind			
	FR-wa	FR-ca	FR-wa	FR-ca	FR-wa	FR-ca	Mihaylov	simple
Comparison.Concession	0.	0.3529	0.2857	0.4211	0.	0.0260	**0.2529**	0.1463
Comparison.Contrast	0.9583	0.9501	0.9467	0.9296	0.3623	0.3636	**0.3934**	0.3559
Contingency.Cause.Reason	0.6392	0.7619	0.7619	0.8837	0.6111	0.7059	0.7037	**0.8438**
Contingency.Cause.Result	0.7742	0.7742	0.7097	0.8493	0.6000	0.6957	**0.9167**	0.8462
Contingency.Condition	0.9556	0.9318	0.9438	0.8947	0.9286	0.9804	0.9455	**0.9811**
EntRel	−	−	−	−	−	−	−	−
Expansion.Alt	0.9091	0.8000	0.8571	0.8333	**0.6667**	0.5882	**0.6667**	**0.6667**
Expansion.Alt.Chosen alt.	0.	0.7143	0.6250	0.5000	−	−	−	−
Expansion.Conjunction	0.9628	0.9596	0.9587	0.9584	0.9565	0.9535	**0.9650**	0.9585
Expansion.Exception	−	−	−	−	−	−	−	−
Expansion.Instantiation	0.8421	0.9474	1.0000	1.0000	0.6667	**0.8571**	0.8000	0.8000
Expansion.Restatement	0.	0.	0.5000	0.	0.	0.	**0.5000**	0.5000
Temporal.Async.Precedence	0.9592	0.9143	0.9143	0.9000	0.9333	0.8434	**0.9620**	0.9487
Temporal.Async.Succession	0.7407	0.7765	0.8352	0.6526	0.8037	0.8148	0.8522	**0.8739**
Temporal.Synchrony	0.7613	0.8098	0.8214	0.6821	0.5524	0.5913	**0.6838**	0.6296
**Overall**	0.8756	0.8907	0.8460	0.8753	0.7230	0.7279	**0.7820**	0.7766

Results for each sense label for Explicit discourse relations on English datasets expressed in *F*_1_-scores using the CoNLL 2016 Shared Task methodology.

Table 9 shows per-sense results for all of the other discourse relation types (Implicit, AltLex, and EntRel) on English datasets. Predicting non-Explicit discourse relations seems to be a substantially more difficult problem. All systems completely fail to recognize 6 sense labels, even on the validation and test datasets. This is not unusual for sense labels with only a few samples, but there should be enough training samples for senses Comparison.Contrast and Temporal.Asynchronous.Precedence. This suggests that the target concept for these senses seems to be unsuitable for current systems, and thus a completely new approach is needed. The system by Mihaylov and Frank (2016) [14] substantially outperforms our models at Expansion.Conjunction and Expansion.Instantiation. For other senses, our model at word level (FR-wa) achieves slightly better or comparable results. Overall low performance for all systems suggests that much more research and different approaches are needed for classifying non-Explicit discourse relations in English.

**Table 9:** Per-sense results for non- Explicit discourse relations on English datasets.

Sense	valid		test		blind			
	FR-wa	FR-ca	FR-wa	FR-ca	FR-wa	FR-ca	Mihaylov	simple
Comparison.Concession	0.	0.	0.	0.	0.	0.	0.	0.
Comparison.Contrast	0.	0.0233	0.	0.	0.	**0.0625**	0.	0.
Contingency.Cause.Reason	0.3099	0.2805	0.2634	0.2576	0.2292	0.1786	0.2136	**0.2604**
Contingency.Cause.Result	0.0984	0.0615	0.1273	0.1062	**0.2500**	0.1860	0.1818	0.2385
Contingency.Condition	−	−	−	−	−	−	−	−
EntRel	0.5368	0.4993	0.4300	0.3781	0.5356	0.4912	**0.5424**	0.4479
Expansion.Alt	−	−	−	−	0.	0.	0.	0.
Expansion.Alt.Chosen alt.	0.	0.	0.	0.	−	−	−	−
Expansion.Conjunction	0.1870	0.3222	0.1747	0.1616	0.2182	0.0320	**0.3444**	0.2320
Expansion.Exception	−	−	−	−	−	−	−	−
Expansion.Instantiation	0.3000	0.	0.1961	0.0270	0.1667	0.0800	**0.2807**	0.1538
Expansion.Restatement	0.	0.2383	0.	0.1107	0.	0.1810	**0.1963**	0.0619
Temporal.Async.Precedence	0.0714	0.	0.2000	0.	0.	0.	0.	0.
Temporal.Async.Succession	0.	0.	0.	0.	−	−	−	−
Temporal.Synchrony	0.	0.	0.	0.	0.	0.	0.	0.
**Overall**	0.3447	0.3420	0.2646	0.2430	0.3415	0.3139	**0.3451**	0.2680

Results for each sense label for Implicit, AltLex, and EntRel discourse relations on English datasets expressed in *F*_1_-scores using the CoNLL 2016 Shared Task methodology.

## Conclusion

In this paper, we move away from hand-engineering and designing a system specifically for a given language, and present a novel system for sense classification of shallow discourse relations (FR system). In contrast to existing systems, we only need to train a single model for all types and senses of discourse relations (no differences in handling Explicit and Implicit discourse relations), perform no preprocessing of its input text spans, nor use any feature engineering or external resources, not even pre-trained word embeddings. The core component of FR system is our novel generalization of the focused RNNs layer, the first multi-dimensional RNN-attention mechanism for generating text/argument embeddings.

The most important characteristic of FR system is the end-to-end trainable approach, that is language-independent with respect to its inputs and architecture, and can be used almost out-of-the-box on any language, label set, and level of input representation. We have confirmed this by successfully applying almost the same model hyper-parameters on two substantially different languages, but also by providing its input at the word and even character levels. It is true, that the model needs to be trained on labeled datasets for each language, but it does not need any language-specific features, pipelines, or resources. It is interesting to note, that attempts at fine-tuning the model hyper-parameters for different setups and introducing pre-trained word embeddings did not substantially improve its performance. On the contrary, disabling either the simple data augmentation technique or weight sharing degraded it.

By following the official task formulation, datasets and methodology of the CoNLL 2016 Shared Task [8], we compared FR system with winning systems and strong baselines on Chinese and English, two substantially different languages. It improved 2.5% over existing best overall results on the Chinese blind dataset (with 0.7477 *F*_1_-score), but did not fall a lot behind state-of-the-art on English blind dataset (with 0.5170 *F*_1_-score). This lack behind state-of-the-art was expected, given that English is the most explored language with advanced language technologies, and systems carefully designed for it can outperform our generic approach. However, significantly lower performance scores of all systems for English than for Chinese indicate, that sense classification on English is much more difficult than on Chinese and *F*_1_-scores are highly affected by differences in grammar, sense labels, and their distribution. This difference is also observed for FR system, that does not achieve comparable *F*_1_-scores on both languages. Because its performance depends on the language, we can not claim it is completely language-independent. This drop in performance when switching to another language could be mitigated by adding language-specific features, external resources, or additional information.

Automated discourse parsing and analysis, especially sense classification of Implicit discourse relations, is a crucial next step in natural language processing. Even though the theoretical grounds for this linguistic phenomena are not fully understood, our single language-independent neural network model is capable of learning the necessary concepts for sense classification without manual feature-engineering efforts and external resources. Furthermore, it is likely that larger amounts of training data or removal of less-frequent sense labels would improve its performance to a practical level.
